# Using age difference and sex similarity to detect evidence of sibling influence on criminal offending

**DOI:** 10.1017/S0033291720003724

**Published:** 2022-07

**Authors:** Janne Mikkonen, Jukka Savolainen, Mikko Aaltonen, Pekka Martikainen

**Affiliations:** 1 Population Research Unit, Faculty of Social Sciences, University of Helsinki, Helsinki, Finland; 2 Department of Criminology & Criminal Justice and Department of Sociology, Wayne State University, Detroit, Michigan, USA; 3 Institute of Criminology and Legal Policy, University of Helsinki, Helsinki, Finland; 4 Law School, University of Eastern Finland, Joensuu, Finland; 5 Department of Public Health Sciences, Stockholm University, Center for Health Equity Studies (CHESS), Stockholm, Sweden; 6 The Max Planck Institute for Demographic Research, Rostock, Germany

**Keywords:** Administrative data, age difference, crime, peer effect, sex similarity, siblings

## Abstract

**Background:**

Sibling resemblance in crime may be due to genetic relatedness, shared environment, and/or the interpersonal influence of siblings on each other. This latter process can be understood as a type of ‘peer effect’ in that it is based on social learning between individuals occupying the same status in the social system (family). Building on prior research, we hypothesized that sibling pairs that resemble peer relationships the most, i.e., same-sex siblings close in age, exhibit the most sibling resemblance in crime.

**Methods:**

Drawing on administrative microdata covering Finnish children born in 1985–97, we examined 213 911 sibling pairs, observing the recorded criminality of each sibling between ages 11 and 20. We estimated multivariate regression models controlling for individual and family characteristics, and employed fixed-effects models to analyze the temporal co-occurrence of sibling delinquency.

**Results:**

Among younger siblings with a criminal older sibling, the adjusted prevalence estimates of criminal offending decreased from 32 to 25% as the age differences increased from less than 13 months to 25–28 months. The prevalence leveled off at 23% when age difference reached 37–40 months or more. These effects were statistically significant only among same-sex sibling pairs (*p* < 0.001), with clear evidence of contemporaneous offending among siblings with minimal age difference.

**Conclusions:**

Same-sex siblings very close in age stand out as having the highest sibling resemblance in crime. This finding suggests that a meaningful share of sibling similarity in criminal offending is due to a process akin to peer influence, typically flowing from the older to the younger sibling.

## Introduction

The assumption that peers influence behavior is among the most widely studied topics in social science (e.g. Hoffman, Sussman, Unger, & Valente, 2006; McGloin & Thomas, 2019; Sacerdote, 2011). However, because selection into peer groups is not random but reflects shared individual and environmental characteristics (e.g. Burt, McGue, & Iacono, 2009), it is difficult to establish the causal effect of peer associations. A recent review of the criminological literature takes a pessimistic view to this methodological challenge: ‘Given our reliance on observational data, the simple fact is *we will never be able to identify the true causal peer effect*’ (McGloin & Thomas, 2019, p. 251, emphasis added).

On a more optimistic note, the econometric literature features at least two studies demonstrating the criminogenic impact of exposure to antisocial peers under *quasi-experimental* conditions. Murphy's (2019) research exploited a natural experiment generated by a policy change aimed at increasing the number of soldiers enlisting in the US Army. To boost its recruiting class, the Army started to issue ‘morality waivers’ to admit offenders with misdemeanor and felony convictions. Murphy found that, among soldiers with no prior criminal record, those assigned to units with higher concentrations of convicts were more likely to engage in misconduct than those with less exposure to criminal peers. In further support, these violations tended to occur in the same month as the acts of misconduct by the morality-waivered peers. Using a similar quasi-random cohort design, Stevenson (2017) found that exposure to high-risk peers while serving time at a juvenile correctional facility had a large impact on post-release criminal offending.

As innovative as those types of studies are, they are limited to *institutional settings* where the dosage of peer exposure may be particularly intense given the lack of access to alternative social circles and nearly constant contact in close quarters. Causal evidence from these special circumstances may not generalize to everyday life. A more adequate test of peer influence, as understood in the theoretical literature, should focus on *interpersonal associations that occur in non-institutional settings*, such as among children living at home and spending unsupervised time in local neighborhoods.

In the course of human development, most of us form relationships with individuals we have not chosen to befriend, yet with whom we interact much the same way as we do with our closest friends. These individuals are known as *siblings*. It is not for us to choose whether we have a brother or a sister, how many of them we will have, and if they are older or younger than we are. Yet it is equally clear that once a sibling has been introduced into our lives, the nature of the relationship shares important similarities with the peers we choose. During our formative years, we spend much of our free time in the company of our siblings: we play with them, we learn skills and attitudes from them, and, to a varying degree, we become embedded in our siblings' social network of friends. Thus, although sibling relationships are in many ways different from non-familial peer associations, *the basic processes of social learning that occur among siblings is similar to those that occur among friends, schoolmates, and other peers* (Ardelt & Day, 2002; Kelly *et al*. 2011; Needle *et al*. 1986). In particular, the processes of social influence that occur among peers are more likely to apply to siblings that are *close in age* and of *the same sex* (Dunifon, Fomby, & Musick, 2017; Rowe & Gulley, 1992). Recognizing that (a) the peer-likeness of a sibling relationship varies by age difference and sex similarity, and that (b) both of those sibling characteristics are exogenous to the sibling relationship, makes it possible to detect the share of sibling resemblance in crime that is due to a social transmission process akin to peer influence.

Thus, in order to observe evidence of peer influence in a natural setting, the present study considers older (full) siblings as special types of peers. Although younger siblings have no choice as to the sex and age of their older siblings, there are two other sources of confounding in the association: genetic relatedness and shared environment. The overall sibling resemblance in delinquency due to these factors has been documented extensively in previous studies (Beaver, 2013; Frisell, Lichtenstein, & Långström, 2011; Lauritsen, 1993; Van de Rakt, Nieuwbeerta, & Apel, 2009). Crucially for our argument, genetic relatedness does not vary by *age difference*. Siblings of different age may experience somewhat different family environments (Conger & Conger, 1994; Jenkins, Rasbash, & O'Connor, 2003; McCall, 1983; Plomin & Daniels, 2011), but most aspects of family environments, such as parenting style or socioeconomic status, can be assumed to be similar. Capitalizing on this insight, the present study utilizes information about the *age difference and sex similarity between siblings* as a way to examine the peer effect of having a criminal older sibling on the criminality of the younger one. To the extent the criminality of the older sibling affects the criminality of the younger sibling (or *vice versa*), independent of the influence of genetic relatedness and shared environment, this association should be particularly strong when the age difference between the siblings is small. In addition, given the high degree of gender homophily in child and adolescent peer associations (McPherson, Smith-Lovin, & Cook, 2001), we expect the between-sibling similarity in criminal offending to be even stronger among *same-sex* sibling pairs who are close in age.

The age-difference hypothesis of the sibling effect on criminal behavior has been examined in two prior studies. Using administrative microdata from Sweden, Kendler, Morris, Lönn, Sundquist, and Sundquist (2014) discovered that the sibling resemblance for the risk of violent crime was stronger in pairs closer in age. In contradiction to our theoretical expectation, the observed patterns were similar regardless of the sex composition of the sibling pairs. Based on total population data, this study covered a wide range of birth cohorts and age groups in the analysis (individuals born in 1951–1991, followed up for criminality from 1973 to 2011 regardless of birth year). Also, as noted, the study was limited to violent crime, and ignored other forms of criminal offending. Another study from the Netherlands considered all types of criminal offending (mean age at the end of follow-up: 27.3) but did not show evidence of age difference moderating sibling similarity in offending (Beijers, Bijleveld, van de Weijer, & Liefbroer, 2017). The study was based on a relatively small number of sibling pairs (<1000) and was therefore limited to crude age difference cutoffs (2½, 3, and 4 years).

Our research can be understood as both a replication and an extension of earlier research. We use Finnish administrative data on the most recent birth cohorts to come of age (children born in 1985–1997), with information on social characteristics and criminal behavior of the siblings and their biological parents. Our analysis differs from prior studies by focusing on criminal offending at ages 11–20 and by using a fine-grained classification of age difference that is not tied to a specific functional form. The chosen age range covers the typical age of onset for criminal careers. These are also ages when the potential for sibling influence is arguably at its strongest due to co-residence, shared school experience, and overlap in the participation in leisure time activities in the local environment. Moreover, following Murphy (2019, see above), we supplement the two previous studies by analyzing the temporal co-occurrence of offending behavior among siblings. It is consistent with theories of peer influence to expect co-offending as well as contemporaneous offending (Warr, 2002).

In sum, in order to detect evidence of interpersonal transmission of criminal behavior between siblings, we investigated three interrelated hypotheses:

Hypothesis 1: The smaller the age difference between the siblings, the stronger the association between the criminality of the older and younger sibling.

Because evidence from a single study is never sufficient for establishing a scientific fact, we think it is important to see if this basic result replicates when using a more comprehensive measure of criminal behavior, a more recent cohort, a narrower age range, and using a dataset from a different national context. As noted by Kendler *et al*. (2014), estimates based on violent criminal behavior may *underestimate* sibling influence, given the evidence that environmental influences are more important for non-aggressive rule-breaking than aggressive antisocial behavior (Burt, 2009).

Hypothesis 2: *The impact of age difference is stronger among same-sex sibling pairs*.

Although Kendler *et al*. (2014) did not find support for this hypothesis, we think it is consistent with the idea of a peer process to expect this. Children and youth are more likely to socialize with individuals of the same gender. We would expect a gendered sibling effect to be particularly prominent during the adolescent years due to greater gender homophily of peer association in those ages.

Hypothesis 3: *The smaller the age difference, the more likely it is for the younger sibling to engage in criminal offending contemporaneously with the older sibling.*

If the assumed social transmission of criminal behavior operates via shared social networks and overlapping routines, it is logical to expect such processes to result in criminal behavior within close temporal proximity.

## Method

### Participants

Similar to other Nordic countries, Finland has a system of interlinked administrative records known as population registries (Haukka, 2004; Lyngstad & Skardhamar, 2011). Statistics Finland delivered the original, anonymized data file (permission TK-53-525-11) that comprised all Finnish children born in 1985–1997 and whose mother lived in Finland in at least one of the years between 2000 and 2015 (*n* = 845 556 persons). The data included identification numbers linking the children to their biological parents.

For the purposes of the present investigation, we identified pairs of full siblings and the two first-born full siblings of larger families (*n* = 231 269 sibling pairs), excluding twins and triplets due to the lack of age difference between them. To ensure the validity of our measurement of criminal offending (see below), we excluded from the analysis sibling pairs who were not resident in Finland at ages 11–20 (*n* = 11 408). To permit accurate measures of parental criminality and family socio-demographic characteristics, we excluded sibling pairs whose parents were not alive or who did not live with at least one of their parents at age 11 (*n* = 6626). The final study sample included 213 911 sibling pairs.

### Key measures

The data in the current study were provided by Statistics Finland, the nation's central repository of administrative data collections. The data on crime and criminal justice outcomes are based on police records. Data on offenses reported to the police (1996–2017) were used to measure criminal behavior. Individuals appear in the record if they were either arrested or named as a suspect in a criminal offense, excluding minor traffic offences.

To ensure comparable measures between the siblings, the criminality of the older and younger sibling was observed over the same developmental period, between ages 11 and 20. Offending behavior was measured as a simple dichotomy indicating the presence of at least one recorded criminal act during this age range. In the analysis examining *contemporaneous* offending, we focused on the years the younger sibling was 11–20 years old regardless of the age of the older sibling. Here, both older and younger sibling offending was measured separately for each year of age as a time-variant dichotomy.

Age difference was measured in bins of 4 months with ‘9–13 months’ as the smallest and ‘48 months or more’ as the largest category. We chose this classification as a trade-off between detail and power.

### Control variables

We included several control variables in the *time-invariant* analysis on overall offending at ages 11–20. Measured in *the year the younger sibling was 11 years of age* (unless otherwise indicated), these covariates are: sex composition of the sibling pair; parents' co-residence (both/mother/father); co-residence of the older sibling and younger siblings (older sibling, no other/older sibling + other/other only/none); geographical urban–rural classification of the residential area (the official 7-grade geographical classification system by the Finnish Environmental Institute); residential instability (times moved at ages 11–20); mother's and father's age at the time the older sibling was born; the criminal record of each parent (offenses reported to the police when the younger sibling was aged 11–20); the highest educational attainment among the parents (higher tertiary, 17+ years/lower tertiary, 14–16 years/upper-secondary, 12 years/comprehensive school only, 9 years); and the unemployment status of each parent (unemployed for more than 6 months). Table 1 shows the distributions of the control variables and their associations with younger and older sibling offending.

**Table 1.** Distribution of the study population and the prevalence of younger and older sibling crime by control variables (*n* = 213 911)

Covariate^a^	Classification	%/Mean	% younger sibling crime at ages 11–20	% older sibling crime at ages 11–20
Sexes of the sibling pair	Both boys	26.1	20.0	18.5
	Both girls	23.9	10.2	8.3
	Boy (o) – Girl (y)	25.0	10.6	18.0
	Girl (o) – Boy (y)	25.0	20.0	8.9
Coresident parents	Both	77.8	12.5	11.1
	Mother	19.4	24.6	22.2
	Father	2.8	27.9	23.2
Coresident older sibling/other siblings	Older sibling, no other	48.7	14.4	12.4
	Older sibling + other	48.0	15.6	13.8
	Other only	1.3	26.0	28.9
	None	2.1	21.4	23.9
Urban–rural classification^b^	Inner urban	20.6	17.8	15.9
	Outer urban	29.8	16.3	14.0
	Peri-urban	13.2	14.7	12.9
	Local centers in rural areas	6.2	15.1	13.2
	Rural areas close to urban	8.9	13.8	12.0
	Rural heartland	14.1	12.8	11.8
	Sparsely populated rural	7.2	12.0	11.6
Times moved at ages 11–20	Continuous	0.9	NA	NA
Mother's age at first birth	Continuous	26.9	NA	NA
Father's age at first birth	Continuous	29.2	NA	NA
Mother crime at ages 11–20	No	96.0	14.5	12.8
	Yes	4.0	34.2	31.8
Father crime at ages 11–20	No	87.7	13.5	11.9
	Yes	12.3	27.8	25.5
Highest parental education	Higher tertiary	16.7	8.8	7.7
	Lower tertiary	38.4	12.7	10.9
	Secondary	40.0	18.8	16.9
	Basic	4.9	29.1	26.8
Mother unemployed	No	94.0	14.7	13.0
	Yes	6.0	25.1	22.8
Father unemployed	No	95.0	14.6	12.9
	Yes	5.0	28.2	25.8
Overall		100.0	15.3	13.5

a Measured in the year the younger sibling was 11 years old if not otherwise mentioned.

b The official geographical classification system of the Finnish Environmental Institute.

The analysis of contemporaneous offending automatically adjusted for all time-constant confounders; however, we further adjusted for potential time-varying confounding by parents' co-residence, co-residence of the older sibling and younger siblings and parents' unemployment. These were measured separately for each year of age, using the same classifications as in the time-invariant analysis (see above).

### Analytical procedure

We examined the first hypothesis by estimating a binary logistic regression model that featured a two-way interaction term between sibling age difference and the criminality of the older sibling as well as all control variables. Based on the parameters of this model, we predicted the probability of crime for each index person (younger sibling) under each level of age difference and older sibling criminality while keeping the control variables at their observed values. These population-level predicted probabilities of crime according to age difference and older sibling crime were averaged out from the individual-level predictions (Muller & MacLehose, 2014; Williams, 2012). In the case of the second hypothesis, we followed a similar procedure but included a *three-way interaction* term between age difference, older sibling crime, and sex composition, presenting the predicted probabilities separately according to the sex composition of the sibling pair. We tested the global statistical significance of age difference–older sibling crime interaction on the additive probability scale (VanderWeele & Knol, 2014).

To investigate the third hypothesis, regarding contemporaneous offending, we estimated a *linear probability model* with individual-level fixed effects for the years the younger sibling was aged 11–20. Because this analysis examines within-individual variation, it eliminates all time-constant individual-level confounding by default (Gunasekara, Richardson, Carter, & Blakely, 2014). However, as a downside, the results are driven by the younger siblings whose older sibling committed at least one crime in the years of interest (*n* = 31 831). As the first step, we estimated a model that included an interaction term between the time-constant sibling age difference and the time-varying older sibling crime, modeling age as a set of dummies. In the subsequent model, we adjusted also for the other time-varying confounders. Standard errors were clustered at the individual level to account for heteroscedasticity and autocorrelation. We conducted all the analyses with Stata, version 15.1 (StataCorp, 2017).

## Results

Table 2 shows the unadjusted differences in the prevalence of younger sibling criminality at ages 11–20 according to age difference and older sibling criminality. Overall, 32% of younger siblings with a criminal older sibling had a criminal record themselves, compared to only around 13% among those without a criminal older sibling. In other words, having an older sibling with a criminal record more than doubles the younger sibling's risk of having one. Regardless of older sibling criminality, younger sibling offending was most common when sibling age difference was less than 13 months: 46% for younger siblings with older sibling offending and around 19% for those without. A slightly higher share of younger siblings has a criminal record.

**Table 2.** Age difference distribution (%) of the study sample and the prevalence of younger sibling crime at ages 11–20 by age difference and older sibling crime (*n* = 213 911)

Age difference (months)	%	Prevalence of younger sibling crime (%)		Difference^a^
		Older sibling crime: no	Older sibling crime: yes	
<13	1.4	18.7	46.0	27.3
13–16	8.2	15.2	39.2	24.0
17–20	13.7	13.0	34.9	21.9
21–24	15.2	12.2	32.6	20.4
25–28	12.4	12.0	30.4	18.4
29–32	10.0	12.3	30.9	18.6
33–36	8.6	12.0	29.9	17.9
37–40	6.3	12.7	27.3	14.7
41–44	4.7	12.8	28.1	15.3
45–48	3.9	12.6	27.7	15.1
>48	15.7	12.4	27.9	15.5
Total	100.0	12.7	31.8	19.1

a Difference in the prevalence of crime between younger siblings with and without older sibling crime.

**Fig. 1.**
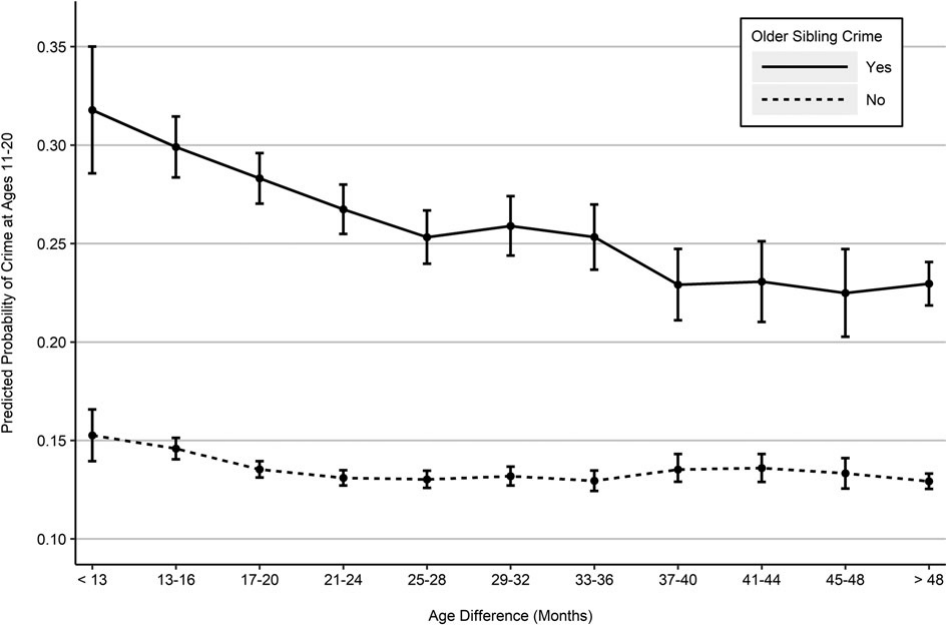
Predicted probabilities of crime at ages 11–20 by older sibling crime and sibling age difference (*n* = 213 911), adjusted for all control variables; global significance of the additive interaction: *p* < 0.001.

The moderating role of age difference in the association between older and younger sibling offending became even clearer when adjusting for all control variables (Fig. 1; see Table 1 for a list of control variables and Table A1 for data). For younger siblings with a criminal older sibling, the estimated prevalence of offending decreased linearly from less than 13 months of age difference (32%) to 25–28 months of age difference (25%) and stabilized when the age difference was 37–40 months or larger (23%). Among adolescents without a criminal older sibling, only a small increase in crime was observed among those with an age difference of 13–16 months or less than 13 months. Overall, small age difference and older sibling offending were associated with a larger than additive risk of younger sibling offending (*p* < 0.001). As a sensitivity check, we restricted the analysis to those living with their older sibling at age 11 and who did not have other siblings. This increased the uncertainty of estimation, but had no distinguishable effect on the shape of the association or the statistical significance of the interaction (result not shown). To ensure that our age difference classification correctly captures the shape of the association, we estimated a generalized additive model with a continuous age difference between 9 and 121 months (Fig. A1). The predicted probabilities did not depart from the ones shown in Fig. 1.

**Fig. 2.**
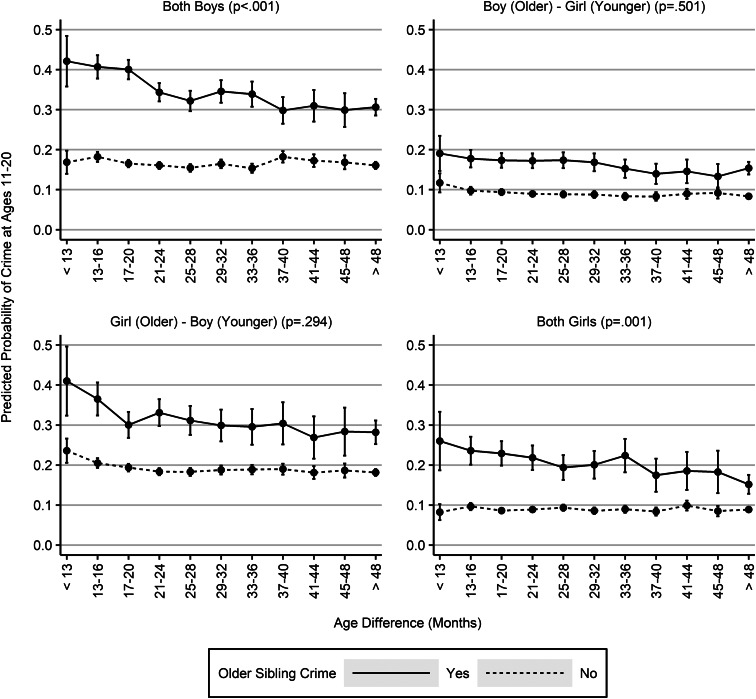
Adjusted predicted probabilities of crime at ages 11–20 by older sibling crime, sibling age difference and the sex combination of the sibling pair (*n* = 213 911), adjusted for all control variables; global *p* values for the additive interaction between age difference and older sibling crime in parentheses.

We repeated the analyses for each sex combination of sibling pairs, again adjusting for all control variables. As shown in Fig. 2, and consistent with Hypothesis 2, the evidence of moderation by age difference was limited to brother–brother (*p* < 0.001) and sister–sister pairs (*p* = 0.001). There was no statistically significant moderation among sex-incongruent sibling pairs (Fig. 2; see Table A2 for data). Because the difference between significant and non-significant coefficients may not itself be statistically significant, we tested whether the interaction between age difference and older sibling crime differed between same-sex and opposite-sex pairs by means of a three-way interaction. This test produced a *p* value of 0.001, indicating a statistically significant difference. Given that the visual inspection of the pattern associated with sibling pairs featuring an older sister and a younger brother suggests some evidence of moderation, we reproduced this analysis with a smaller number of age difference categories – all the adjacent categories, except for the open-ended category, were combined – to increase statistical power. Consequently, the positive results on same-sex brother and sister pairs became even more pronounced (results not shown), and the difference between same-sex and opposite-sex sibling pairs remained statistically significant (*p* = 0.005).

**Fig. 3.**
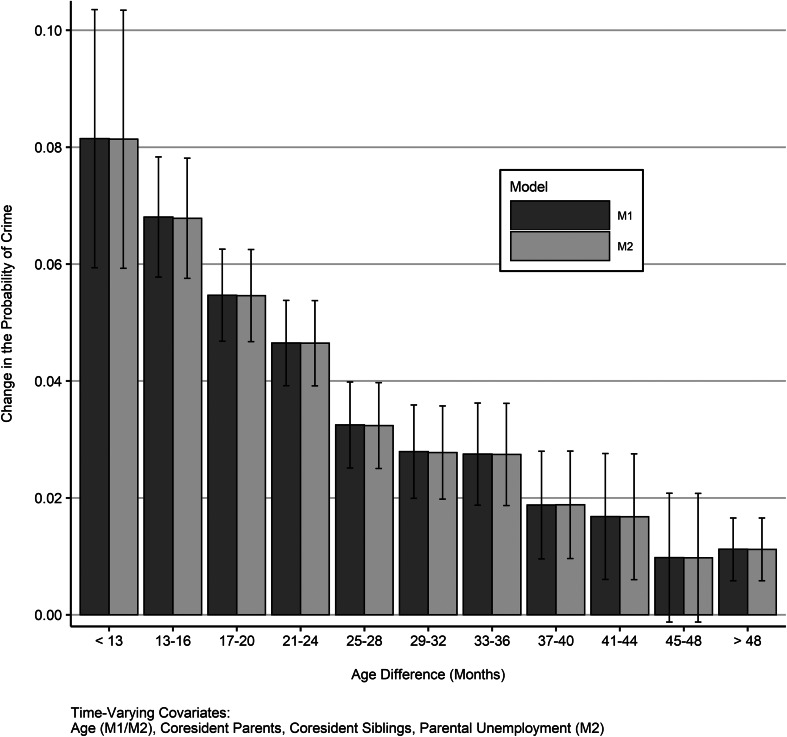
Change in the probability of crime if older sibling committed a crime in the same year, separately according to sibling age difference (*n* = 213 911); global significance of the additive interaction in both models: *p* < 0.001.

Our fixed-effects analysis of contemporaneous offending estimated the absolute increase in younger sibling offending if the older sibling engaged in criminal offending *in the same year* (Fig. 3; see Table A3 for data). We observed, once again, strong moderation by age difference that operated in an almost dose–response manner. Among siblings with an age difference of less than 13 months, the estimated increase in crime was around 8 percentage points, while the equivalent increase was 3 percentage points for sibling pairs with an age difference of 25–28 months, and only 1 percentage point for pairs whose age difference was more than 48 months. All models were adjusted for the age of the younger sibling. Further adjusting for time-variant confounding by parents' co-residence, co-residence of the older sibling and younger siblings, and parents' unemployment did not affect the estimates.

## Discussion

We framed this study as an investigation of *peer influence* on criminal offending. We made the argument that, in childhood and adolescence, same-sex siblings who are very close in age are especially likely to interact as peers. Based on this assumption, we proposed three hypotheses about sibling resemblance in criminality. In support of our first hypothesis, we found the age difference of siblings to moderate the positive association between the criminality of the siblings. The association was particularly strong when age difference was minimal (less than 2 years). This non-linear pattern is consistent with peer influence as we would expect siblings who are very close in age to interact with each other more frequently and intensely, and we would not expect age difference to matter once it gets so large as to render peer processes implausible. This may explain why the study by Beijers *et al*. (2017) failed to detect evidence of moderation by age difference. They used 2.5 years as the cut-off for the smallest age difference.

In further support of peer influence, we found that moderation by age difference was limited to same-sex sibling pairs; we did not find robust evidence of moderation by age difference among sex-incongruent sibling pairs. A parsimonious and theoretically sound explanation of these patterns is that same-sex siblings are more likely to spend time together outside the family setting. Note that this result contradicts Kendler *et al*. (2014) who found evidence of moderation across each sex combination of sibling pairs. We can think of two factors as possible explanations of this discrepancy. First, our data were focused on adolescence whereas Kendler *et al*. (2014) tracked criminal convictions across a wider spectrum of the life course, including mature adulthood. This difference could produce different results regarding the salience of sibling sex given that gender homophily in peer groups declines dramatically in post-adolescent ages (McPherson *et al*. 2001). Second, Kendler *et al*.'s (2014) research was limited to violent offending, whereas our study used a broader measure of any criminal offending other than minor traffic violations. As noted, violent offending is more heritable than non-violent offending, which suggests more room for environmental effects in more general measures of crime.

Our third and final test of the peer mechanism focused on the timing of criminal offending. In support of the hypothesis, we found small age difference to increase the likelihood of the younger sibling committing a criminal offense in the same year as the older sibling. Although this evidence does not directly demonstrate co-offending, it is consistent with that assumption as well as a more generalized peer process, such as imitation or contagion (Murphy, 2019).

Outside the literature on peer effects in criminal offending, previous research has produced mixed results on whether narrow birth spacing *in itself* increases the risk of adverse social and health-related outcomes in offspring (Barclay & Kolk, 2017; Buckles & Munnich, 2012; Conde-Agudelo, Rosas-Bermudez, Castaño, & Norton, 2012). In our study, we found an association between the smallest possible age difference and offending even among those younger siblings whose older sibling had *not* committed crimes at the same age. However, most of this association disappeared controlling for the socio-demographic characteristics of parents. Moreover, the age difference slope among those without a criminal older sibling was never as clear as it was among those who had one. Thus, while the criminality of the older sibling and small age difference have independent effects on younger sibling criminality, our results show that their interactive effect is larger than the additive effect of the two factors.

In terms of theoretical framing, our investigation assumed the sibling effect to flow from older to younger sibling. This assumption is consistent with prior research suggesting that exposure to older delinquent peers is an important pathway to criminal behavior (Harding, 2009). However, it is reasonable to assume *reciprocity* in the observed associations (Whiteman, Jensen, & McHale, 2017) – especially among siblings who are close in age. Kendler *et al*. (2014) addressed the direction of causality in the sibling effect by comparing situations where the older *v.* the younger sibling was the first to have received the criminal conviction. Their analysis showed evidence of stronger transmission from older to younger sibling, but small age difference was associated with larger sibling similarity in offending regardless of which sibling was convicted first.

To supplement our main analysis, we examined the temporal ordering of older and younger sibling offending in our data. As reported in Table A4, narrow age difference was associated with an increased likelihood that the onset of criminal offending by younger sibling occurred either in the same year or prior 2 years before the onset of criminal offending by the older sibling. These results suggest that the direction of sibling influence becomes increasingly ambiguous as the age difference gets small. Nevertheless, we find that, regardless of the age difference, it was more typical for the older siblings to engage in criminal offending prior to the younger sibling. This suggests that most of the effect does indeed flow from the older to the younger sibling. Note, also, that because these data are based on crimes known to the police, they are a poor indicator of the true age of onset in criminal offending. It is conceivable that, even in situations where the younger sibling's criminality is detected first, the older sibling's (undetected) involvement in delinquency may have exerted influence.

## Limitations

Administrative data obviate many threats to the validity of survey-based longitudinal data, such as non-response, attrition, and preferential reporting (Lyngstad & Skardhamar, 2011). However, the process whereby offending behavior gets recorded by the police may vary by the characteristics of the offender and/or the area in which he or she lives (MacDonald & Fagan, 2019). It is unclear if any such factors introduce bias in our estimates. It is conceivable that having a criminal older brother will make teachers and other agents of social control pay more attention to the younger sibling. However, when it comes to policing, we expect this kind of process to be less salient, especially in Finland where police presence is relatively low (Eurostat, 2019) and the practice of patrolling high-crime areas is virtually non-existent. In this national context, it does not seem realistic to assume that adolescents become targeted by police officers simply by virtue of their siblings' prior contact with the law.

There may be other threats to the validity of our conclusions. It is plausible that parents adjust the way they parent a younger sibling based on their experiences with the older ones. If they do not like the way the first child behaves, parents may reexamine their methods and decide to do things differently with the second child. Although this would affect the second child regardless of the age difference, an argument can be made that adjustment in parenting will be more profound with increased age difference. Parents may not have enough information about the first child to modify their parenting style regarding the younger sibling if the age difference is only 1–2 years. Under this assumption, the patterns observed in our research could be explained by more substantial parenting changes in situations where the age difference is large. In the absence of measures of parenting, we were unable to directly examine this possibility with our data. Note, however, that changes in parenting are unlikely to explain our results on contemporaneous offending, thus significantly diminishing the likelihood that changes in parenting can bias our results.

## Conclusion

Due to systematic selection, it is notoriously difficult to identify causal effects of environmental factors on human behavior. Although it is sometimes possible to devise randomized field experiments to study social processes, a number of theoretically compelling social factors do not lend themselves to such manipulation. Relevant examples include parenthood, romantic relationships, and friendship formation. There are examples in the peer effects literature of quasi-experimental studies that have exploited exogenous variation in peer exposure in institutional settings. This kind of research offers persuasive evidence of peer influence, but under very special circumstances. Our research adds to this literature by presenting evidence of quasi-peer effects as they emerge over time in natural settings of everyday life.

Using sibling age difference and sex similarity as identifying assumptions, we found evidence of sibling peer influence on criminal behavior. We encourage scholars to expand this approach to additional behavioral outcomes where peer processes are assumed to exert a strong influence. The relevant domains could include research on educational outcomes and sexual and reproductive behavior. Prior research by Kendler, Ohlsson, Sundquist, and Sundquist (2013) applied this analytic strategy to substance misuse. Overall, studies that have used such designs demonstrate that older siblings are an important source of influence on younger same-sex siblings *who are very close in age*. Interventions that target the older sibling could have strong spillover effects on the younger one. Perhaps this potential mechanism could be studied further and – if validated – exploited in the development of effective prevention programs to tackle antisocial behavior.

## Supplementary material

For supplementary material accompanying this paper visit https://doi.org/10.1017/S0033291720003724. click here to view supplementary material
