# Supplementary material for: Using age difference and sex similarity to detect evidence of sibling influence on criminal offending
Source: Psychol Med. 2020 Oct 21;52(10):1892–900. doi: 10.1017/S0033291720003724 (PMC9343217; doi:10.1017/S0033291720003724)

Table A.1. Data for Figure 1; predicted probabilities (P) and their 95% confidence intervals (CI)

			95% CI	
Older sibling crime	Age difference (months)	P	Lower	Upper
No	< 13	0.153	0.139	0.166
	13-16	0.146	0.140	0.151
	17-20	0.135	0.131	0.139
	21-24	0.131	0.127	0.135
	25-28	0.130	0.126	0.135
	29-32	0.132	0.127	0.137
	33-36	0.129	0.124	0.135
	37-40	0.135	0.129	0.143
	41-44	0.136	0.129	0.143
	45-48	0.133	0.126	0.141
	> 48	0.129	0.125	0.133
Yes	< 13	0.318	0.286	0.350
	13-16	0.299	0.284	0.314
	17-20	0.283	0.270	0.296
	21-24	0.267	0.255	0.280
	25-28	0.253	0.240	0.267
	29-32	0.259	0.244	0.274
	33-36	0.253	0.237	0.270
	37-40	0.229	0.211	0.247
	41-44	0.231	0.210	0.251
	45-48	0.225	0.203	0.247
	> 48	0.230	0.219	0.241

Table A.2. Data for Figure 2; predicted probabilities (P) and their 95% confidence intervals (CI)

Older sibling crime	Age difference (months)	Both boys			Both girls			Boy (o) - Girl (y)			Girl (o) -Boy (y)		
			95% CI			95% CI			95% CI			95% CI	
		P	Lower	Upper	P	Lower	Upper	P	Lower	Upper	P	Lower	Upper
No	< 13	0.169	0.140	0.197	0.082	0.062	0.102	0.117	0.093	0.141	0.236	0.206	0.266
	13-16	0.182	0.170	0.194	0.096	0.087	0.105	0.098	0.088	0.107	0.205	0.193	0.217
	17-20	0.165	0.156	0.175	0.086	0.079	0.093	0.094	0.087	0.101	0.194	0.184	0.203
	21-24	0.161	0.152	0.169	0.089	0.082	0.095	0.089	0.082	0.096	0.183	0.175	0.192
	25-28	0.154	0.145	0.164	0.093	0.086	0.101	0.088	0.081	0.096	0.183	0.173	0.193
	29-32	0.165	0.154	0.175	0.086	0.078	0.094	0.088	0.079	0.096	0.188	0.177	0.199
	33-36	0.154	0.142	0.165	0.089	0.081	0.098	0.083	0.075	0.092	0.189	0.177	0.200
	37-40	0.182	0.168	0.196	0.084	0.074	0.094	0.083	0.072	0.094	0.190	0.176	0.203
	41-44	0.172	0.157	0.188	0.099	0.086	0.111	0.090	0.077	0.102	0.181	0.165	0.197
	45-48	0.168	0.151	0.186	0.085	0.072	0.098	0.092	0.078	0.106	0.186	0.169	0.204
	> 48	0.161	0.152	0.169	0.089	0.082	0.095	0.083	0.077	0.090	0.181	0.173	0.190
Yes	< 13	0.421	0.358	0.484	0.260	0.187	0.333	0.191	0.147	0.235	0.410	0.323	0.496
	13-16	0.407	0.378	0.436	0.236	0.201	0.271	0.178	0.156	0.199	0.365	0.324	0.406
	17-20	0.400	0.376	0.425	0.229	0.199	0.259	0.173	0.155	0.191	0.300	0.268	0.333
	21-24	0.344	0.321	0.366	0.218	0.188	0.249	0.172	0.154	0.191	0.331	0.297	0.365
	25-28	0.322	0.296	0.347	0.194	0.163	0.225	0.174	0.154	0.193	0.311	0.275	0.348
	29-32	0.346	0.317	0.374	0.201	0.166	0.235	0.168	0.146	0.191	0.299	0.259	0.339
	33-36	0.339	0.307	0.370	0.224	0.182	0.265	0.153	0.130	0.175	0.295	0.251	0.340
	37-40	0.298	0.265	0.332	0.175	0.133	0.216	0.140	0.114	0.165	0.304	0.252	0.357
	41-44	0.310	0.270	0.349	0.185	0.138	0.233	0.146	0.116	0.175	0.269	0.216	0.322
	45-48	0.299	0.257	0.341	0.183	0.130	0.236	0.133	0.102	0.164	0.284	0.224	0.343
	> 48	0.306	0.285	0.327	0.152	0.128	0.175	0.154	0.138	0.169	0.282	0.253	0.311

Table A.3. Data for Figure 3; predicted probabilities (P) and their 95% confidence intervals (CI)

			95% CI	
Age difference (months)	Model[1]	P	Lower	Upper
< 13	M1	0.081	0.059	0.104
13-16	M1	0.068	0.058	0.078
17-20	M1	0.055	0.047	0.063
21-24	M1	0.046	0.039	0.054
25-28	M1	0.032	0.025	0.040
29-32	M1	0.028	0.020	0.036
33-36	M1	0.027	0.019	0.036
37-40	M1	0.019	0.010	0.028
41-44	M1	0.017	0.006	0.028
45-48	M1	0.010	-0.001	0.021
> 48	M1	0.011	0.006	0.017
< 13	M2	0.081	0.059	0.103
13-16	M2	0.068	0.058	0.078
17-20	M2	0.055	0.047	0.062
21-24	M2	0.046	0.039	0.054
25-28	M2	0.032	0.025	0.040
29-32	M2	0.028	0.020	0.036
33-36	M2	0.027	0.019	0.036
37-40	M2	0.019	0.010	0.028
41-44	M2	0.017	0.006	0.028
45-48	M2	0.010	-0.001	0.021
> 48	M2	0.011	0.006	0.017

[1]Time-varying covariates: age (M1/M2), coresident parents, coresident siblings, parental unemployment

Table A.4. The temporal ordering of earliest police-reported offending among older and younger siblings (%) according to age difference among sibling pairs where at least one sibling committed a crime in 1996-2017 (n=65,663)

Age difference (months)	Older first	Same year	Younger first	Younger did not commit	Older did not commit
< 13	13.4	6.0	12.1	32.6	35.9
13-16	11.6	4.6	8.9	34.0	41.0
17-20	10.7	3.8	7.1	36.5	41.8
21-24	10.5	3.3	7.0	37.1	42.0
25-28	10.0	2.8	6.5	40.0	40.8
29-32	10.4	2.7	6.4	39.0	41.5
33-36	11.3	2.9	5.4	39.7	40.8
37-40	10.5	2.1	5.6	40.7	41.1
41-44	11.5	2.0	5.4	40.7	40.4
45-48	12.7	1.5	5.2	41.9	38.7
> 48	14.4	1.4	3.5	44.6	36.1
Total	11.4	2.9	6.3	39.2	40.3

Figure A.1. Predicted probabilities of crime at ages 11-20 by older sibling crime and sibling age difference (n=213,911); based on a generalized additive model (fitting method: restricted maximum likelihood) that features a smooth function of age difference (measured at the precision of one month) and adjusts for all control variables

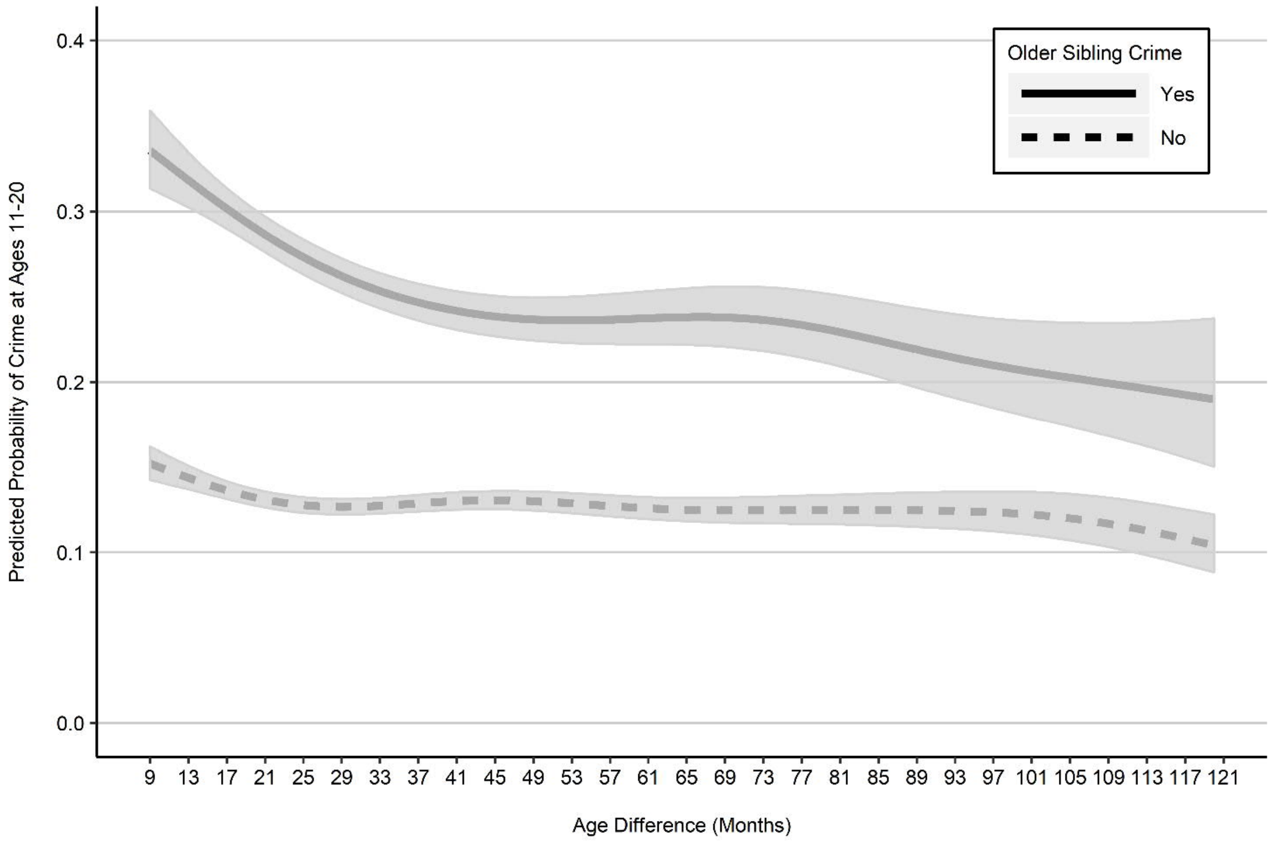

Supplement: Supplementary file 1 [file S0033291720003724sup001.pdf]
